# Cinnamaldehyde Downregulation of Sept9 Inhibits Glioma Progression through Suppressing Hif-1*α* via the Pi3k/Akt Signaling Pathway

**DOI:** 10.1155/2022/6530934

**Published:** 2022-01-19

**Authors:** Zhiwen Wang, Changfeng Wang, Jieping Fu, Ruen Liu, Xinhui Zhou

**Affiliations:** ^1^Department of Neurosurgery, Jiangxi Provincial People's Hospital Affiliated to Nanchang University, Nanchang 330006, China; ^2^Department of Neurosurgery, Yichun Second People's Hospital, Yichun 336000, China; ^3^Department of Neurosurgery, Peking University People's Hospital, Beijing, 100044, China; ^4^Department of Neurosurgery, The First Affiliated Hospital of Nanchang University, Nanchang 330006, China

## Abstract

**Purpose:**

Cinnamaldehyde (CA) is the main ingredient in cinnamon, and it has been proven to have an inhibitory effect on many different tumor types. However, it lacks effect on glioma. This paper explores the effect CA has on glioma cells U87 and U251 at the cellular and molecular levels.

**Methods:**

The relationship between Hif-1*α* and Sept9 was found by CGGA. Cell Viability Assay (CCK8) was made to detect the proliferation ability. The scratch experiment and the transwell experiment were applied to the migration and invasion ability. Annexin V-FITC/PI were used to detect the cell apoptosis. Western blotting was used to determine the specified protein level.

**Results:**

Cell proliferation assay results revealed CA to inhibit the proliferation of glioma cells in a dose-dependent manner. It promoted apoptosis for upregulating the expression of Bax and downregulating the expression of Bcl-2. Wound Healing Assay and transwell test found CA to have anti-invasion ability and that it upregulated the expression of E-cadherin and downregulated the expressions of MMP-2 and MMP-9. The molecular mechanism was studied from a tumor microenvironment (TME) perspective. Pi3k inhibitor (LY294002) was used for interfering with cells, and the results found CA to demonstrate a similar effect. Hif-1*α* and Sept9 expressions were inhibited, and Akt and p-Akt were also inhibited. By using CoCl_2_ to make hypoxia, CA was discovered to inhibit the high expression of Hif-1*α* and Sept9, demonstrating a correlation with the Pi3k/Akt pathway. It is suggested that the mechanism of Sept9 under hypoxia regulation can be realized through the Pi3k/Akt pathway.

**Conclusions:**

This study proves for the first time that CA is an effective drug for inhibiting the proliferation of glioma through Sept9 and reveals Sept9 to be related to the Pi3k/Akt pathway in terms of tumor microenvironment, providing a molecular basis for the further study of CA in glioma treatment.

## 1. Introduction

Glioma is the most common tumor that is found in the brain's nervous system. Treatments for it include chemotherapy, radiotherapy, surgery, targeted therapy, and stem cell therapy. However, it can easily relapse, it has a high mortality rate, and the prognosis is poor [1]. In recent years, the pharmacological effects of herbaceous plants have made significant progress, including tea polyphenols [2] and indirubin [3], which can effectively inhibit the progress of tumor cells. It has been proven that CA can inhibit colon cancer [4, 5], breast cancer [6], small cell lung cancer [7], and bone marrow-derived suppressor cells (MDSCs) [8] among others [9], but a lack of research has been conducted relating to the treatment of glioma with CA.

CA can inhibit expression of vascular endothelial growth factor (VEGF), reduce neovascularization and cancer cell proliferation, and promote cancer cell apoptosis through tumor hypoxia microenvironmental factor Hif-1*α* [10, 11]. Therefore, research on the target of Hif-1*α* has currently become a topic of great interest. It has been shown that Sept9 can bind and stabilize Hif-1*α*, promote its transcriptional expression, and promote angiogenesis and tumor growth [12, 13]. By using Chinese Glioma Genome Atlas (CGGA) gene correlation analysis, it was discovered the significant positive correlation between Sept9 and Hif-1*α*. It has been proven that cinnamaldehyde can inhibit the growth and invasiveness of cancer cells through the Pi3k/Akt pathway. In this paper, experiments were conducted on this basis in order to confirm that CA can inhibit the expression of Sept9 and Hif-1*α* through the Pi3k/Akt pathway, inhibit the tumor microenvironment, and restrict tumor growth.

## 2. Materials and Methods

### 2.1. Chemicals, Reagents, and Antibodies

CA was purchased from MedChemExpress LLC China (purity 99%). This was dissolved in dimethylsulfoxide (DMSO) at a stock solution (200 *μ*g/ml) and stored at -80°C. Dulbecco's modified Eagle's medium (DMEM) and fetal bovine serum (FBS) were purchased from Servicebio (Wuhan, China), and antibodies against Akt, p-Akt, Bax, MMP-2, MMP-9, Hif-1*α*, Sept9, and *β*-actin were purchased from Servicebio (Wuhan, China). LC3B was purchased from Bioss (Beijing China), Bcl-2 was purchased from Cell Signal Technology (Massachusetts, USA), rabbit anti-human antibody against Pi3k inhibitor LY 294002 was purchased from Cell Signal Technology (Massachusetts, USA), CoCl_2_ was purchased from Aladdin (Shanghai, China), CCK8 kit was purchased from Biosharp Life Sciences (Beijing, China), and Annexin V conjugated to fluorescein-isothiocyanate (Annexin V-FITC) apoptosis detection kit was purchased from Beyotime (Beijing, China). All other chemicals that were used in the experiment were of the highest purity grade available.

### 2.2. Cell Culture

The glioma cell lines (U87, U251) were purchased from the Chinese Academy of Medical Sciences (Beijing, China) and cultured in DMEM that was supplemented with 10% fetal bovine serum (FBS) in an incubator that contained 5% CO_2_ at 37°C.

### 2.3. Cell Viability Assay (CCK8 Analysis)

3 × 10^3^ cells were uniformly cultured in 96-well plates for 12, 24, and 48 hours and were then treated with 0, 2, 4, 8, and 16 *μ*g/ml of CA. 10 *μ*l of CCK8 was added and incubation at 37°C continued for 30 minutes. The solution was then detected with a microplate reader, and the absorbance was checked at 490 nm.

### 2.4. Cell Apoptosis Assay

The Annexin V-FITC/PI apoptosis detection kit was used. Glioma cells were inoculated in a 6-well plate and treated with CA (0, 4, and 8 *μ*g/ml) for 24 hours. The cells were harvested following trypsin digestion. After the cells were washed with cold phosphate buffer (PBS) twice, the cells were centrifuged for five minutes (1000g), and the supernatants were removed to allow for cell collection. PBS heavy suspension count: 5 ~ 10 × 10^4^ cells were centrifuged for five minutes (1000g) and suspended in 195 *μ*l binding buffer. Annexin V-FITC (5 *μ*l) and 10 *μ*l PI were added to the mix before incubation for 30~60 min in the dark at room temperature. The PBS was then washed twice, and the quenching solution was added before the film was observed under the fluorescence microscope.

### 2.5. Wound Healing Assay

U87 and U251 cells were cultured in 6-well plates. When they grew to approximately 85% confluence, they were scratched with a new 200 *μ*l pipette tip and washed twice using PBS. The cells were then treated with 4 and 8 *μ*g/ml of CA for 24 and 48 hours. Pictures were taken under a microscope. ImageJ software was used for the collection of images and to quantify the gap distance.

### 2.6. Invasion Assay

Transwell membrane filter inserts were used (pore size, 8 *μ*m; Costar, Corning, NY, USA) in 24-well dishes. U87 and U251 cells were pretreated with 4 *μ*g/ml and 8 *μ*g/ml CA for 24 hours and then inoculated with approximately 1 × 10^4^ cells in 200 *μ*l of serum-free medium in the upper chamber and 600 *μ*l medium containing 15% bovine serum in the lower chamber. They were incubated at 37°C with 5% CO_2_ for 24 hours and then fixed in 4% paraformaldehyde for 30 minutes and stained in PBS with 0.05% crystal violet for 30 minutes. The cells were gently removed from the upper part of the filter using a cotton swab, the filter was allowed to dry naturally, and the cells from the lower part of the filter were checked and counted under a microscope.

### 2.7. Clinical Tissue Samples

Tumor tissue samples and adjacent normal brain tissue samples were obtained from eight patients who were undergoing glioma surgery, and the samples were rapidly preserved at Jiangxi Provincial People's Hospital Affiliated to Nanchang University (Jiangxi, China) from October 2019 to December 2020. None of the glioma patients received radiotherapy or chemotherapy prior to surgery while they were hospitalized. Each participant in this study provided written informed consent. The clinical characteristics of the patients were collected, including age, sex, and pathological findings (including WHO grade, immunohistochemical information, and genetic information). The study protocol was approved by the Ethics Committee of Jiangxi Provincial People's Hospital Affiliated to Nanchang University. The glioma and nonneoplastic brain tissue that were collected were fixed with formalin, embedded in paraffin, and cut into 5 *μ*m thick sections for immunohistological analysis. The tissue was then frozen in liquid nitrogen and held at −80°C until calculation.

### 2.8. Western Blot Analysis

U87 and U251 cells were cultured and treated in 6-well plates. They were cleaved in an ice bath in the RIPA buffer for 30 minutes and centrifuged at 12,000g at 4°C for 15 minutes. The extracted supernatant was stored at -80°C until analysis. The protein concentration was determined using the BCA method (Beyotime). An equal volume of protein was loaded onto a 10% SDS-polyacrylamide gel for electrophoresis and was then transferred by electrophoresis to a polyvinylidene fluoride (PVDF) membrane (Millipore, Boston, MA, USA), which was blocked with 5% bovine serum albumin (BSA) at room temperature overnight at 4°C and then incubated with the primary antibodies against MMP-9 (1 : 1,000), MMP-2 (1 : 1,000), E-cadherin, Bax (1 : 1,000), Bcl-2 (1 : 1,000), Akt (1 : 1,000), Hif-1*α* (1 : 1,000), *β*-actin (1 : 2,000), Sept9 (1 : 800), LC3B (1 : 1,000), and p-Akt (1 : 500). After being washed with a mixture of Tris-buffered saline and Tween-20 (TBST) three times (5 min/time), difluorescent antibody diluted at 1 : 2,000 was added at room temperature for 1 hour. SPECTRA MAX SoftMax Pro analysis software was used for the detection of the OD signal intensity of each band on the film. *β*-Actin was used as load control and normalization.

### 2.9. Statistical Analysis

All data are presented as means ± standard deviation (SD). The experiment was repeated a minimum of three times, and an independent *t*-test was used for making a comparison between the two groups. One-way analysis of variance was used for making comparative analysis between multiple groups with SPSS 26.0 software. *p* < 0.05 was considered to be statistically significant.

## 3. Results

### 3.1. There Was a High Expression and High Correlation of Sept9 and Hif-1*α* in Gliomas

**Figure 1:**
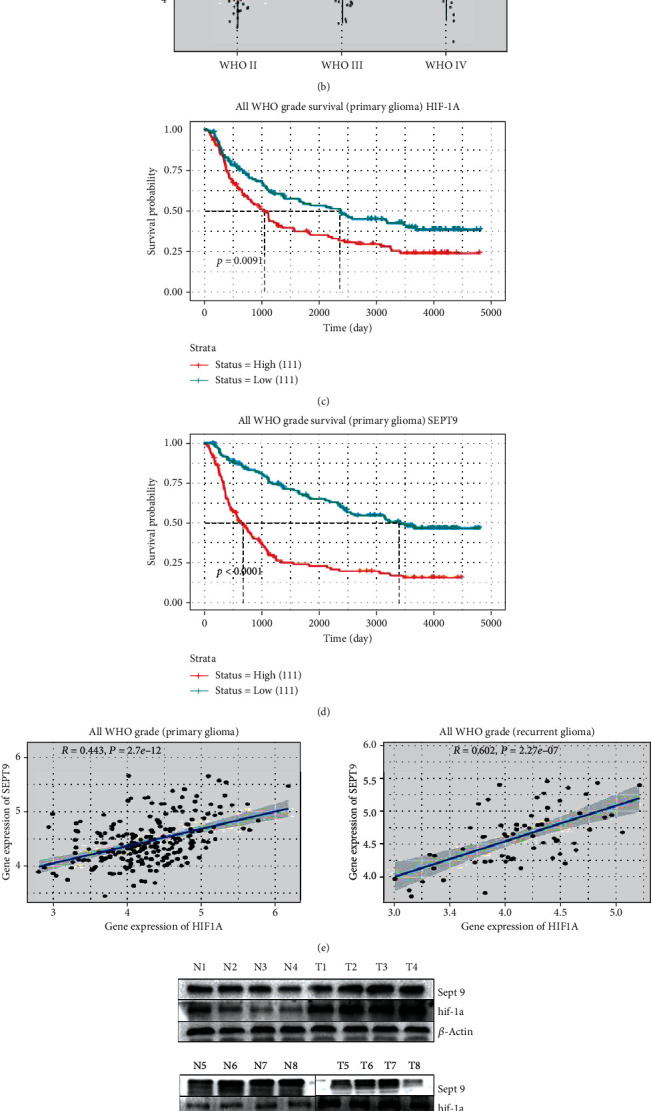
The expression of Hif-1*α* and Sept9 in tissues. CGGA database analysis shows the following: (a, b) the expression of Hif-1*α* and Sept9 in WHO grade of glioma (orange: WHO II, green: WHO III, and blue: WHO IV); (c, d) the relationship between Hif-1*α* and Sept9 survival rate in primary glioma patients; (e) the correlation between Hif-1*α* and Sept9 in primary and recurrent gliomas; (f, g) the expression of HIF-1*α* and Sept9 in tumor tissues and adjacent normal tissues of eight glioma patients.

The high expression of Sept9 and Hif-1*α* in CGGA datasets was detected, showing a positive correlation with WHO grade (Figures 1(a) and 1(b)). There was also a positive correlation between them and patient survival rate. It is suggested that glioma patients with low expressions of both have a better overall survival rate (Figures 1(c) and 1(d)). The correlation analysis of Sept9 and Hif-1*α* found there to be a high correlation between Sept9 and Hif-1*α* in both primary and recurrent tumors with WHO grade (Figure 1(e)). Western blotting analysis also confirmed high expressions of Sept9 and Hif-1*α* in glioma tissues and adjacent normal tissues in eight patients, conforming to WHO grade (Figures 1(f) and 1(g)). The above results demonstrate that a key role is played by Sept9 in the regulation of glioma progression and is closely related to Hif-1*α*.

### 3.2. CA Inhibited the Growth and Invasion of Glioma Cells (U87 and U251)

**Figure 2:**
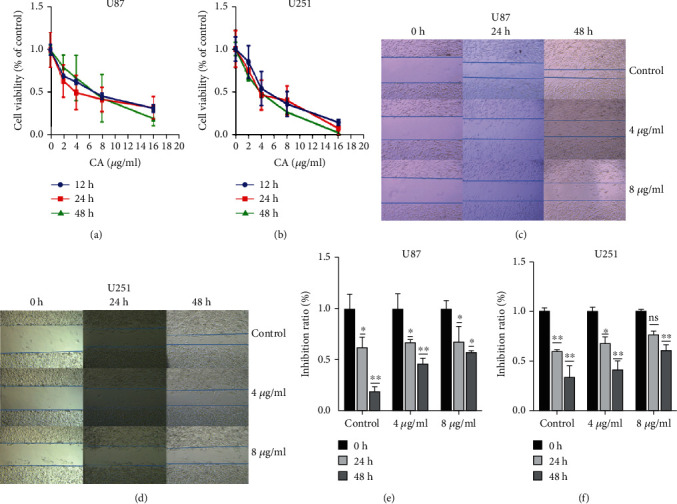
The effects of CA on the cell viability and migration of U87 and U251 cells. (a, b) U87 and U251 cells were treated with control, 2 *μ*g/ml, 4 *μ*g/ml, 8 *μ*g/ml, and 16 *μ*g/ml of CA for 12, 24, and 48 hours. Cell viability rate was measured using CCK8 assay. (c, d) Statistical analysis of scratches. Wound healing analysis was used for determining the migration of U87 and U251 cells for 24 and 48 hours. (e, f) The results of a minimum of three independent trials are presented as mean ± standard deviation (SD). ^∗^*p* < 0.05 and ^∗∗^*p* < 0.01 compared to the control group.

This study examined whether CA can inhibit the proliferation of glioma cells in vitro. Following treatment with 0, 2, 4, 8, and 16 *μ*g/ml of CA for 12, 24, and 48 hours, the effects of different concentrations of CA on cell viability were observed using the CCK8 method (Figures 2(a) and 2(b)). The cell survival rate following CA intervention was found to be significantly lower than in the control group in a dose-dependent manner (*p* < 0.01), and a certain time-dependent relationship was observed in the world group. In order to clarify the effect CA has on the migration of glioma cells, the inhibition of CA on the migration of U87 and U251 cells was demonstrated through wound healing experiments. Cells were treated with 0.4 and 8 *μ*g/ml, and then, images of them were obtained under a microscope after 24 and 48 hours (Figures 2(c) and 2(d)). The results showed that CA inhibited cell migration in a dose-dependent manner (*p* < 0.05) (Figures 2(e) and 2(f)).

### 3.3. CA Promotes Apoptosis and Inhibits Cell Invasion

**Figure 3:**
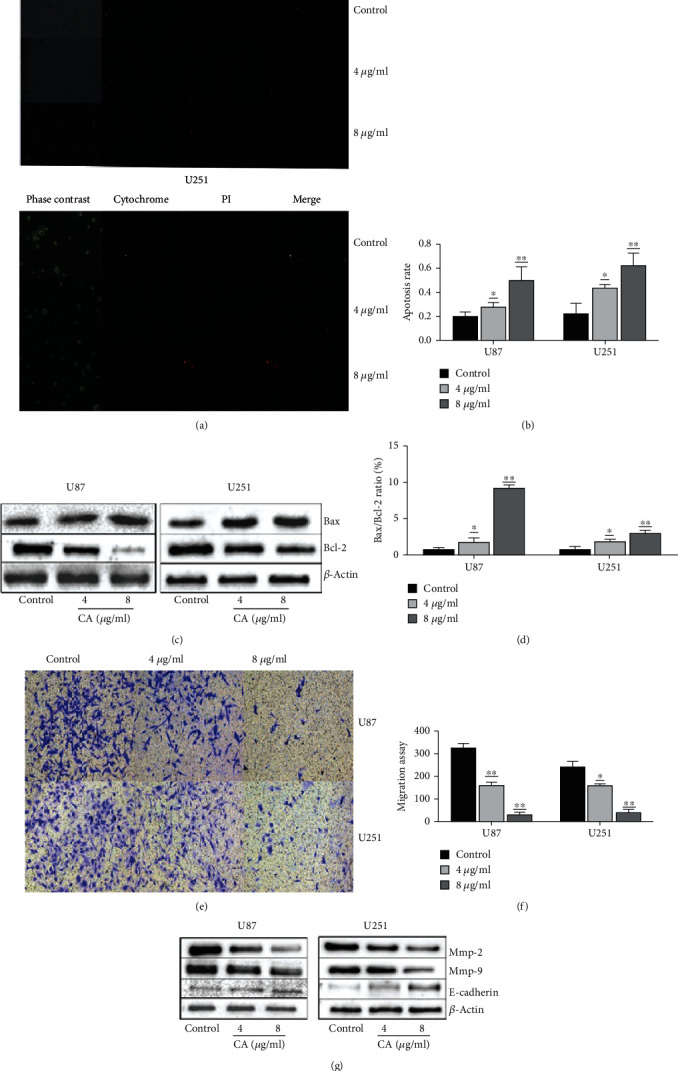
The effects of CA on cell apoptosis and invasive ability of U87 and U251 cells. (a) The cells were inoculated in 6-well plates and treated with CA (4 *μ*g/ml and 8 *μ*g/ml) for 24 hours. Annexin V-FITC/PI apoptosis was detected and photographed under a fluorescence microscope. (b) Statistical results of fluorescence microscope images from the apoptosis experiment. (c) CA induces apoptosis through the regulation of apoptosis-related genes. U87 and U251 cells were treated with 0, 4, and 8 *μ*g/mL CA for 24 hours. Western blot was used for detecting the expressions of Bcl-2 and Bax, and *β*-actin was the control. (d) The expression levels of Bax and Bcl-2 were measured. The effect of CA was then evaluated using Bax/Bcl-2 ratio. (e) Following treatment with different concentrations (4 *μ*g/ml and 8 *μ*g/ml) of CA, the cells migrated through pores of 8 *μ*m in diameter to the lower lumen within 24 hours, where images were captured using a light microscope (magnification 400x). The white dots are the 8 *μ*m diameter pores of the transwell chamber. (f) Statistical results of invasion experiment. (g) CA suppresses the expressions of MMP-2 and MMP-9 while increasing the expression of E-cadherin. U87 and U251 cells were treated with CA (4 and 8 *μ*g/ml) for 24 hours. The results of a minimum of three independent trials are presented as mean ± SD. ^∗^*p* < 0.05 and ^∗∗^*p* < 0.01 compared to the control group.

To confirm whether CA can induce apoptosis of glioma cells, the effect of CA on glioma cells was detected by Annexin V-FITC/PI double staining. Apoptosis was recorded using a fluorescence microscope and counted (Figures 3(a) and 3(b)). The results demonstrated that CA increased the rate of apoptosis in a dose-dependent manner, and the apoptosis rate of cells treated with 0, 4, and 8 *μ*g/ml CA was significantly higher than the control group (*p* < 0.01). While conducting an exploration of the molecular mechanism of apoptosis induced by CA in human glioma cells, the expression of apoptosis-related proteins induced by 0 *μ*g/ml, 4 *μ*g/ml, and 8 *μ*g/ml CA was detected by Western blotting. Analysis showed the expression of Bax to have increased significantly, whereas the expression of Bcl-2 decreased, which resulted in an increase in Bax/Bcl-2 ratio (Figures 3(c) and 3(d)).

### 3.4. Transwell Chamber Test Was Used for Detecting Whether CA Inhibits the Invasion of Glioma Cells (Figure 3(e))

The results showed that following treatment with CA (0, 4, and 8 *μ*g/ml) for 24 hours, compared to the control group, the number of cells that invaded the lower chamber through holes with a diameter of 8 *μ*m diameter decreased significantly, which indicates CA inhibited the invasion of glioma cells in a dose-dependent manner and that the inhibitory effect on cell invasion was enhanced as CA concentration increased (Figure 3(f)). The expression levels of MMP-2, MMP-9, and E-cadherin were analyzed using Western blotting (Figure 3(g)). CA significantly reduced the expressions of MMP-2 and MMP-9 in a concentration-dependent manner. The expression of E-cadherin was upregulated as the CA dose increased, which is potentially one of the mechanisms for reducing glioma cell invasiveness and adhesion.

### 3.5. CA Inhibited the Expressions of Hif-1*α* and Sept9, While Also Inhibiting the Expressions of Akt and p-Akt

**Figure 4:**
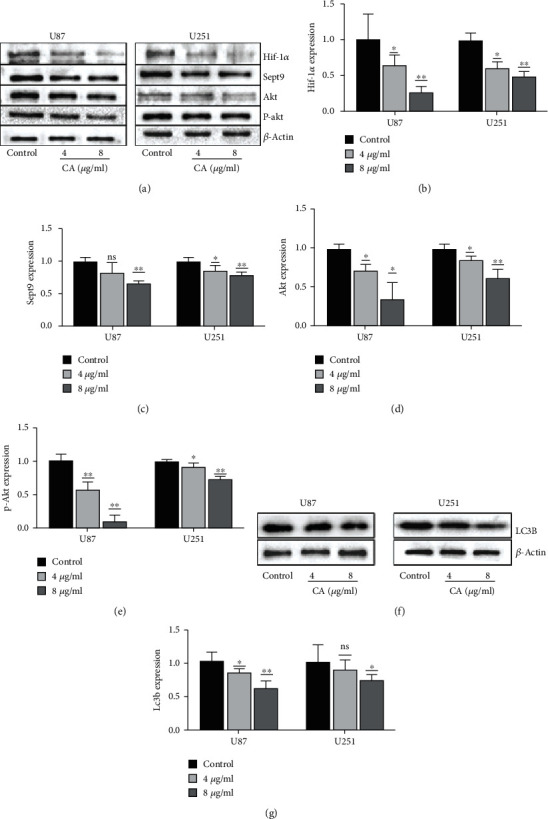
CA inhibits the expression of related proteins. (a) U87 and U251 cells were treated with CA (4 and 8 *μ*g/ml) for 24 hours. The expression levels of Hif-1*α*, Sept9, Akt, and p-Akt were detected by Western blot analysis, and *β*-actin was used as a loading control. (b–e) Statistical results of the expressions of Hif-1*α*, Sept9, Akt, and p-Akt. (f) The expression levels of LC3B were detected using Western blot analysis, and *β*-actin was used as a loading control. The results of a minimum of three independent trials are presented as mean ± SD. ^∗^*p* < 0.05 and ^∗∗^*p* < 0.01 compared to the control group.

Akt is the key factor of the classical signal path Pi3k/Akt. Western blotting was used for detecting the expression of related proteins in human glioma cells induced by 0, 4, and 8 *μ*g/ml CA (Figures 4(a) and 4(f)). The results showed that CA significantly decreased the expressions of Hif-1*α*, Sept9, and LC3B in a concentration-dependent manner (Figures 4(b), 4(c), and 4(g)). At the same time, it also inhibited the expressions of Akt and p-Akt (Figures 4(d) and 4(e)). From the statistical results, it can be seen that when the concentration of CA is 8 *μ*g/ml, the above proteins are statistically significant (*p* < 0.05).

### 3.6. The Effect of CA on the Pi3k/Akt Signal Pathway in Glioma Cells

**Figure 5:**
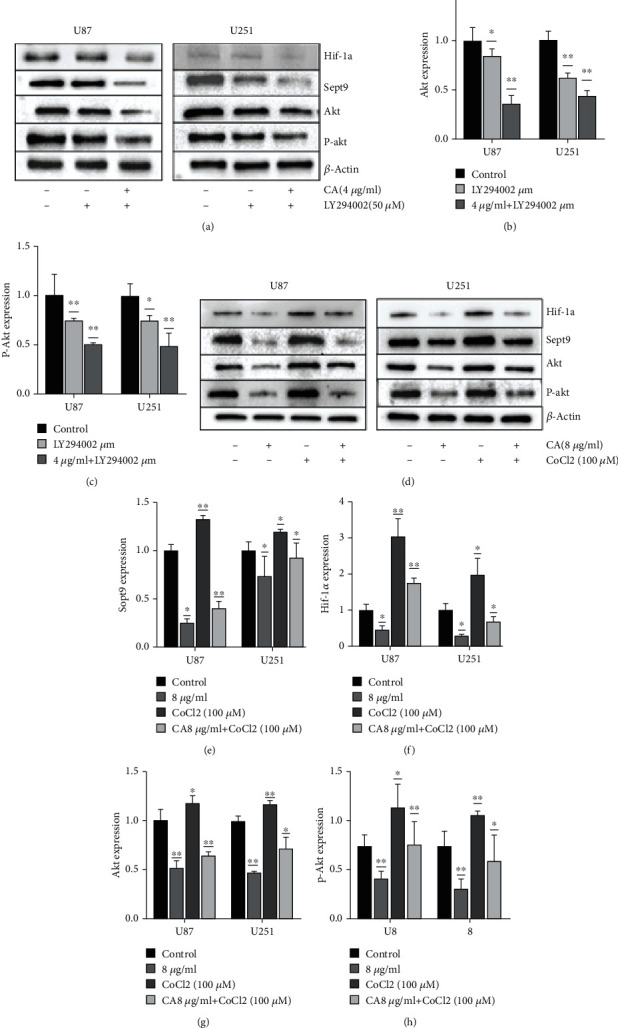
The effects of cinnamaldehyde (CA) on the Pi3k/Akt signaling pathway in U87 and U251 cells. (a) U87 and U251 cells were treated with CA (CA 4 *μ*g/ml and CA 4 *μ*g/ml) and LY294002 (50 *μ*M) for 24 hours. The expression levels of Hif-1*α*, Sept9, Akt, and p-Akt were detected using Western blot analysis, and *β*-actin was used as a loading control. (b, c) The statistical results of the expressions of Akt and p-Akt. (d) U87 and U251 cells were treated with CA (CA 8 *μ*g/ml) and CA 8 *μ*g/ml+CoCl_2_ (100 *μ*M) for 24 hours. The expression levels of Hif-1*α*, Sept9, Akt, and p-Akt were detected using Western blot analysis, and *β*-actin as the control. (e–h) Statistical results of the expressions of Hif-1*α*, Sept9, Akt, and p-Akt. The results of a minimum of three independent trials are presented as mean ± SD. ^∗^*p* < 0.05 and ^∗∗^*p* < 0.01 compared to the control group.

The Pi3k/Akt signaling pathway is involved in the regulation of the biological behavior of cells. To provide further confirmation of the results, LY294002 (50 *μ*M), a specific inhibitor of Pi3k [5], and CA 4 *μ*g/ml were used as positive controls for 24 hours. Western blot analysis showed the effect of CA to be similar to that of LY294002, while it inhibited the expressions of Hif-1*α*, Sept9, Akt, and p-Akt protein (Figures 5(a)–5(c)). To determine whether CA can regulate glioma cell apoptosis through the Pi3k/Akt pathway under the condition of tumor anoxic microenvironment, the tumor anoxic microenvironment was pretreated with CoCl_2_ (100 *μ*M) for 24 hours, while the control group was treated with CA 8 *μ*g/ml. Western blotting showed cobalt chloride to increase the expressions of Hif-1*α*, Sept9, Akt, and p-Akt (Figure 5(d)). Western blotting demonstrated that CA inhibited the expressions of Hif-1 *α*, Sept9, Akt, and p-Akt when 8 *μ*g/ml CA acted on the cells that were treated with CoCl_2_ (*p* < 0.05) (Figures 5(e)–5(h)).

## 4. Discussions

Malignant glioma has long been a problem that is difficult for clinicians. A high mortality rate and poor prognosis are a significant burden to both patients and society. Several methods are used for the treatment of glioma, but due to the characteristics of malignant proliferation, high recurrence, and easy migration, there is a worldwide consensus for studying the pathological mechanism of tumor cells and the way in which tumor progression can be curbed through molecular pathways. The study of tumor microenvironment is currently of great interest in terms of research. Under hypoxia, tumor angiogenesis increases, tumor cell proliferation and migration accelerate, and Hif-1*α* is the most important factor [14]. The Pi3k/Akt/Hif-1*α* pathway has been confirmed through experiments, but finding new target proteins remains the key point [15, 16]. The antitumor effect of many drugs is achieved by blocking this pathway. CA is the main component that can be extracted from traditional herbaceous plants [10]. Several studies on tumors have confirmed the inhibitory effect of CA on tumor growth and migration, but comparatively little glioma research has been conducted [9].

Using the CGGA database, it was discovered that the expression of Hif-1*α* in WHO grade increased as the malignant degree increased and had a positive correlation with patient survival rate. Hif-1*α* is the core factor of anoxic microenvironment and is highly expressed in several different tumors [7, 17]. The mechanism study is mainly reflected in the increase of reactive oxygen species (ROS) in cancer cells for the further promotion of apoptosis [18] and epithelial-mesenchymal transformation (EMT) [18, 19] and to promote the expression of vascular growth factor, including matrix-derived factor-1 (SDF-1), VEGF, and platelet-derived growth factor B (PDGFB), among others [20, 21]. Binding to PD-L1 in tumors limits T cell growth, increases apoptosis, and activates autophagy [19]. Hif-1*α* has many mechanisms that are worthy of deeper exploration.

The Sept9 gene is located on human chromosome 17q25.3, containing 17 exons and being approximately 240 × 103 bp in length. It encodes 15 types of peptide [22] and has the function of recruiting proteins to the cytoplasm [23]. It is also related to the morphological change and transformation of cells. Sept9 has direct involvement in actin dynamics, autophagy, angiogenesis, cell proliferation, cell motility, and microtubule regulation, and it has also been reported that Sept9 is involved in glioblastoma development [24, 25]. Some studies have found Sept9 to be a key factor for the binding and stabilization of Hif-1*α* and that it can increase the transcription of Hif-1*α* and activate Sept9-Hif-1*α*, thereby forming blood vessels and promoting tumor growth [26]. A high expression of Sept9 can effectively inhibit Hif-1*α* ubiquitination and degradation. CGGA gene analysis also found the expression to increase as the degree of malignancy defined by WHO grade increased, and that it had a positive correlation with patient survival rate. There was a correlation between Hif-1*α* and Sept9. Tumor samples and adjacent normal tissues from eight patients with different glioma types were detected by WB, and the expressions were found to be significantly high in WHO grades. This study has explored the changes of Sept9 in U87 and U251 cells treated with CA to become a potential therapeutic target. However, it is unknown which type of cellular signaling pathway it is related to.

CA was used to interfere with U87 and U251, the results showing that it could effectively inhibit proliferation and migration. Annexin V-FITC/PI double staining showed that CA can effectively promote the apoptosis of glioma cells at 4 *μ*g/ml and 8 *μ*g/ml. Western blot apoptotic protein analysis has shown that targeting Bcl-2 family proteins and Bax proteins are common factors of apoptosis induced by many anticancer drugs, and the proportion of Bax/Bcl-2 plays a key role [27]. As CA dose increased, WB showed a high expression of Bax, but a decreased expression of Bcl-2. Bax/Bcl-2 increased significantly as CA concentration increased, and the effect of CA 8 *μ*g/ml was significant. The results of the invasion experiment showed CA could inhibit the invasion of cells. E-cadherin is a member of the cadherin superfamily, and matrix metalloproteinases (MMPs) can degrade extracellular matrix proteins, thereby affecting the invasion and differentiation of cells. With the aforementioned MMPs, the activities of MMP-2 and MMP-9 gelatinases have a close relationship with tumor metastasis [28]. Increasing the concentration of CA led to the expressions of MMP-2 and MMP-9 being inhibited, whereas the expression of E-cadherin was increased.

CA promotes apoptosis while inhibiting the proliferation of glioma cells, but is it related to Hif-1*α* and Sept9? Some literature has proven CA to reduce the expression of VEGF via the Pi3k/Akt pathway [29]. Therefore, an attempt was made to detect the expression of Akt and p-Akt in glioma cells that had been treated with CA. The results showed that the expressions of Hif-1*α*, Sept9, Akt, and p-Akt were inhibited as CA concentration increased. The effect is obvious when CA is 8 *μ*g/ml. At the same time, the autophagy protein LC3B [30] was detected, and the results showed it to be significantly inhibited when CA was 8 *μ*g/ml. Autophagy has a close relationship with anoxic microenvironment [31], but the further relationship requires further study in future experiments.

Further experiments verified the Pi3k/Akt pathway. The Pi3k/Akt pathway has been reported to inhibit apoptosis and facilitate the promotion of cell survival [16]. U87 and U251 cells were treated with LY249002 and CA, and the results showed CA to exhibit a similar effect to LY249002. The statistical results showed the synergistic effect of CA and LY 249003 to be significant. Many reports on the hypoxia model have been made by CoCl_2_ [7, 32, 33]. Hypoxia can facilitate the promotion of the Pi3k/Akt pathway [34, 35]. Therefore, the control group was set up and CA 8 *μ*g/ml and CoCl_2_ (100 *μ*M) were used for interfering with glioma cells in order to extract protein for WB detection. The statistical results showed CoCl_2_ to promote the expressions of Akt and p-Akt, and the expressions of Hif-1*α* and Sept9 increased. However, when CA 8 *μ*g/ml was added with cobalt chloride, CA's inhibitory effect improved, and the expressions of Akt and p-Akt increased slightly. Therefore, it can be speculated that CA inhibits glioma progression via the Pi3k/Akt pathway, and Hif-1*α* and Sept9 exhibit the same trend. However, it is unknown whether Hif-1*α* combines with Sept9 to produce such a synergistic effect, and this requires further confirmation through experiments.

## 5. Conclusions

The results of this study suggest that the high expression of Sept9 in gliomas is highly correlated with Hif-1*α*. This is consistent with previous CGGA statistical results, so the inhibitory effect CA has on glioma cells has a significant relationship with Hif-1*α*. It is also suggested that a decrease in Sept9 expression may be a new target for effectively inhibiting glioma progression. There were some limitations to this experiment, so further experiments are required for verification of the mechanism of CA inhibiting Sept9 in glioma.

## Data Availability

My experimental data, including tables and pictures, were obtained from specific experiments, and the article included the analysis results and pictures of relevant data. The data used to support the findings of this study is available from the corresponding author upon request.
